# Co-expression With Replicating Vector Overcoming Competitive Effects Derived by a Companion Protease Inhibitor in Plants

**DOI:** 10.3389/fpls.2021.699442

**Published:** 2021-06-17

**Authors:** Jiexue Ma, Xiangzhen Ding, Zhiying Li, Sheng Wang

**Affiliations:** ^1^Key Laboratory of Ministry of Education for Protection and Utilization of Special Biological Resources in Western China, Yinchuan, China; ^2^School of Life Science, Ningxia University, Yinchuan, China; ^3^Key Laboratory of Modern Molecular Breeding for Dominant and Special Crops in Ningxia, Yinchuan, China

**Keywords:** protease inhibitor, competitive effects, transient expression, replicating vector, agroinfiltration, *Nicotiana benthamiana*

## Abstract

Plant-based expression platforms are currently gaining acceptance as a viable alternative for the production of recombinant proteins (RPs), but the degradation of RPs by proteases in cells hinders their superb potentials. Co-expression of a protease inhibitor (PI) shows promise as a strategy to prevent RP from proteolytic degradation in plants. However, competitive effects behind the PI-RP co-expression system may mask or obfuscate the *in situ* protective effects of a companion PI. Here, we explored the competitive effects by co-expressing reteplase (rPA) with three unrelated PIs, namely *Nb*PR4, *Hs*TIMP, and *Sl*CYS8, in *Nicotiana benthamiana* leaves. Remarkably, the accumulation of rPA was significantly repressed by each of the three PIs, suggesting that the competitive effects may be common among the PIs. The repression can be attenuated by reducing the PI inoculum dose in the co-inoculation mixtures, showing a negative correlation between the PI abundance of the PI-RP system and competitive effects. Interestingly, when a replicating vector was used to modulate the relative abundance of PI and RP *in vivo*, rPA was still boosted even at the maximal testing dose of PI, indicating that the competitive effects reduced to an ignorable level by this *in vivo* approach. Furthermore, a 7- to 12-fold increase of rPA was achieved, proving that it is a useful way for stimulating the potentials of a companion PI by overcoming competitive effects. And, this approach can be applied to molecular farming for improving the RP yields of plant expression systems.

## Introduction

In recent years, plants are gradually regarded as a viable alternative for producing recombinant proteins (RPs; Shanmugaraj et al., 2020), due to their potential for low energy requirement, reduced animal pathogen contamination risks, and post-translational modifications (Schillberg and Finnern, 2021). The RPs can be essentially produced in plants by either stable transformation or transient expression (Gils et al., 2005). And, the latter approach is more efficient in terms of time consumption as well as batch processing (Kopertekh and Schiemann, 2019). Furthermore, the *Agrobacterium*-mediated methods were established to deliver the transgenes into the host cells for transiently producing RPs in plants (Zhang et al., 2020). When more than one gene should be expressed at the same time, it can be achieved conveniently by mixing the *Agrobacterium* cultures carrying different transgenes just before agroinfiltration *in vitro*. Various RPs have been expressed transiently in plants in recent years, including proteins for therapeutic, diagnostic, research, and industrial applications. Several plant-derived RPs have reached or are close to the market (Fox, 2012; Schillberg and Finnern, 2021), but the relatively low yields of RPs limited more of them to be commercialized (Zischewski et al., 2016; Schillberg and Finnern, 2021).

Proteases are pervasive in all organisms and are the essential regulators in living cells (Schaller, 2004). Hundreds of proteases are encoded by plant genomes and hold crucial functions in controlling protein turnover, regulating development, and responding to defense (Tripathi and Sowdhamini, 2006). It was reported that more than 1,000 protease genes were identified in the *Arabidopsis* genome (Schaller, 2004), and 975 putative proteases were annotated in agroinfiltrated *Nicotiana benthamiana* leaves (Grosse-Holz et al., 2018a). RPs are therefore faced with a diverse and complex proteolytic microenvironment while expressing in plants. Since proteases can degrade RPs whether *in vitro* (Paireder et al., 2017) or *in vivo* (Benchabane et al., 2008), they are the major sponsors for the low accumulation of RPs in cells.

Several approaches have been developed to escort RPs against degradation in plant cells, including subcellular targeting, stabilizing agents, gene knockdown, fusion partners, and co-expression of protease inhibitors (PIs; Mandal et al., 2016). Among them, a companion PI offers at least two significant advantages. First, it may avoid the requirements of adding PIs during protein purification processes and therefore reduce the cost of products (Robert et al., 2016). Second, transiently co-expressing PI has minimal side effects on plant tissues due to temporal and local limitations (Grosse-Holz et al., 2018b).

Some PIs were found to boost RPs accumulation *in planta* by either stable transformation (Komarnytsky et al., 2006; Pillay et al., 2012) or transient expression (Goulet et al., 2012; Robert et al., 2013; Jutras et al., 2016). However, *in vivo* protective effects of PIs on RPs are not as efficient as theoretically expected in the application. Sainsbury et al. (2013) reported that the co-expression of *Sl*CYS8 (a tomato Cys protease inhibitor) with human alpha-1-antichymotrypsin (α1ACT) in plants resulted in a marked decrease in α1ACT accumulation instead of increasing by preventing its degradation by leaf Cys proteases as expected, suggesting an apparent competitive effect between PI and RP during pre- and/or post-translational processes (Sainsbury et al., 2013). They tried to overcome the competitive effect by fusing the *Sl*CYS8 and α1ACT to be one chimeric protein. However, *Sl*CYS8 acts as a stabilizing fusion partner to increase α1ACT levels instead of a protease inhibitor (Sainsbury et al., 2013). There are only a few studies available on this issue since then. Recently, it was shown that the protective effects of PIs dramatically increased when the competitive effects were eliminated by using a non-functional PI as control (Grosse-Holz et al., 2018b). Therefore, the competition may mask the potential of PIs, which causes the protective effects of a companion PI to be largely underestimated in applications.

To address this issue, three PIs, namely *Nb*PR4, *Hs*TIMP, and *Sl*CYS8, that were used previously to increase RP accumulation upon co-expression (Grosse-Holz et al., 2018b), were selected for this study. And, rPA, a truncated human tissue-type plasminogen activator (tPA), was used as a model RP due to instability when expressed in plant tissues (Hidalgo et al., 2017). We explored the competitive effects behind the PI-RP system by co-expressing rPA with those unrelated PIs (belonging to different families of PIs; I25 family for *Sl*CYS8, I43 for *Nb*PR4, and I35 for *Hs*TIMP) in *N. benthamiana* leaves. Subsequently, we demonstrated a negative correlation between the PI abundance and competitive effects by reducing PI inoculum dose in mixtures. Finally, we diminished the competitive effects to an ignorable level by co-expressing a replicating vector with PI.

## Materials and Methods

### Construction of Co-expressed Vectors

**Figure 1:**
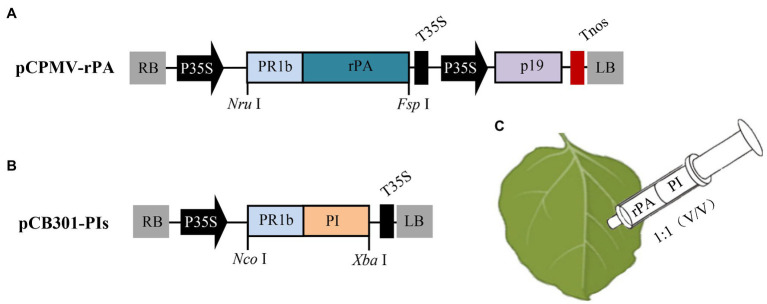
Schematic representation of the construction of the co-expression vectors and the co-agroinfiltration strategy. **(A)** The T-DNA region of pCPMV-rPA construct, a non-replicating rPA expression vector. LB and RB, the left and right T-DNA borders; P35S, CaMV 35S promoter; PR1b, the secretory signal peptide of tobacco pathogenesis-related protein 1b gene; rPA, the *N. benthamiana* codon-optimized rPA gene; T35S, CaMV transcription terminator; p19, the gene of silencing suppressor from TBSV (*Tomato bushy stunt virus*); Tnos, the terminator of nopaline synthase gene. **(B)** The T-DNA region of the pCB301-PI construct, for expressing protease inhibitor (PI). PI, either of *Nb*PR4, *Hs*TIMP, or *Sl*CYS8; *Nru*I, *Fsp*I, *Nco*I, and *Xba*I, restriction enzymes. **(C)** The agroinfiltration strategy for transiently co-expressing rPA with PI in *N. benthamiana*. Bacterial inoculum for the rPA and PI were mixed at an equal volume and infiltrated into leaves *via* a syringe.

The binary plasmid pCB301 (Xiang et al., 1999) was used as the backbone for all constructs. And, *in vitro* DNA synthesis and restriction enzyme-mediated cloning were employed to create the co-expressed constructs. For the construction of the non-replicating rPA expression vector, the CPMV expression cassette (Peyret and Lomonossoff, 2013) was synthesized (GenScript) and cloned into the pCB301 *via Apa*I and *Bam*HI restrict sites to generate pCPMV-EV construct. Then the *N. benthamiana* codon-optimized rPA (Invitrogen) with an N-terminal PR1b signal peptide (GenBank accession no. D90197.1) was synthesized and cloned into pCPMV-EV using *Nru*I and *Fsp*I restriction sites to generate pCPMV-rPA construct (Figure 1A). For plant-based expression of the inhibitors, synthetic sequences of the PIs gene (*Nb*PR4, *Hs*TIMP, or *Sl*CYS8) were separately cloned into plasmid pCBNoX P19 (Dong et al., 2019) using *Nco*I and *Xba*I restriction sites generating pCB301-PIs constructs (Figure 1B). To obtain the replicating rPA expression vector, the rPA expression cassette was cloned into plasmid pJL TRBO-G, a TMV (*Tobacco mosaic virus*)-based vector (Lindbo, 2007), by *Pac*I and *Not*I restriction sites to create the pTMV-rPA construct. Meanwhile, the digested DNA of pJL TRBO-G was made blunt using Klenow fragment and circularized by self-ligation yielding pTMV-EV. After construction, plasmids were transformed into *Agrobacterium tumefaciens* strain GV3101 *via* the freeze-thaw method.

### Transient Co-Expression of rPA With PIs in Plants

**Figure 2:**
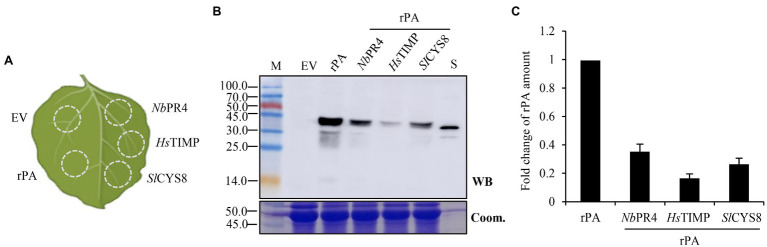
Co-expression of PI with a non-replicating vector (pCPMV-rPA) represses rPA accumulation in plants. **(A)** Schematic representation of the agroinfiltrated zones within a leaf. EV, empty vector, negative control; rPA, rPA alone; *Nb*PR4, co-expression of rPA and *Nb*PR4; *Hs*TIMP, co-expression of rPA and *Hs*TIMP; *Sl*CYS8, co-expression of rPA and *Sl*CYS8. **(B)** Western blot analysis of crude leaf protein homogenate. The Coomassie-stained large subunit of Rubisco was used as a loading control (lower panel) and the polyclonal rabbit anti-tPA antibody was used to immunodetect the recombinant rPA (upper panel). M, molecular weight marker (kDa); S, *E. coli*-expressed rPA, positive control. **(C)** ELISA assay of the relative rPA accumulation following the co-expression of PI. The data are presented as fold change of rPA amount in the absence or presence of PI and are determined from three separate batches of infiltrations. The average value for rPA alone is 69.7 μg/g +/− 0.7.

**Figure 3:**
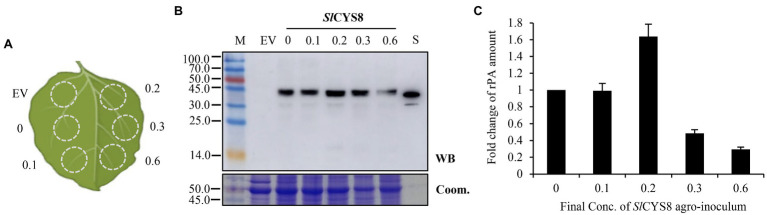
Modulating the dose of *Sl*CYS8 inoculum in mixtures attenuates repression on rPA accumulation. **(A)** Schematic representation of the agroinfiltrated zones within a leaf. The numbers show the final OD_600_ value of the *Sl*CYS8 inoculum in mixtures, while the final value of 0.6 is for the rPA construct. **(B)** Western blot analysis of crude leaf protein homogenate. The Coomassie-stained large subunit of Rubisco was used as a loading control (lower panel) and the polyclonal rabbit anti-tPA antibody was used to immunodetect rPA (upper panel). M, molecular weight marker (kDa); EV, empty vector, negative control; S, *E. coli*-expressed rPA, positive control. **(C)** ELISA assay of the relative rPA accumulation following *Sl*CYS8 co-expression. The data are presented as fold change of rPA amount in the absence or presence of *Sl*CYS8 and are determined from three separate batches of infiltrations. The average value for treatment “0” is 69.2 μg/g +/− 0.9.

The recombinant *A. tumefaciens* were grown in liquid LB media supplying appropriate antibiotics (kanamycin, rifampicin, and gentamycin, 50 μg/ml for each) with gentle agitation for ~24 h at 28°C. Then, the cultures were centrifuged and re-suspended in an appropriate volume of 10 mM MES buffer (pH 5.6, 10 mM MgCl_2_, and 200 μM acetosyringone) to obtain the required density (Optical Density [OD] value at 600 nm, OD_600_; the OD_600_ value 1.2 for rPA; 1.2, 0.6, 0.4, and 0.2 for *Sl*CYS8 separately). For co-expression, the suspensions were mixed in the ratio of 1:1 (V/V; Figure 1C), left in the dark for 2 h of incubation, and then infiltrated into *N. benthamiana* leaves with a syringe. For comparison, the individual mixes were infiltrated into various zones within a leaf (Figures 2A, 3A). In one experiment, 15–20 seedlings and three leaves of each seedling were infiltrated. Three independent experiments were performed.

### Extraction of Proteins From Leaf Tissues

The infiltrated zones of the leaf were sampled with a hole punch at 5 day post-inoculation (dpi) in all experiments, and nine discs from three individual plants were merged per sample. The leaf discs were weighed and then ground in pre-cooled buffer (50 mM Tris-HCl, 100 mM NaCl, 5 mM ethylenediaminetetraacetic acid [EDTA], 0.1% Tween 20, 0.1% protease inhibitors cocktail, pH 8.0) at a ratio of 1:4 (g/ml). The homogenates were clarified in a centrifuge at 4°C (12,000 *g* for 15 min), and the supernatant was collected for later assays.

### Gel Electrophoresis and Western Blot

A total of 10 μl of clarified leaf homogenate was mixed with loading buffer, heated for 5 min, and then separated on a 12% polyacrylamide gel. After electrophoresis, one gel was for Coomassie blue staining, and another was blotted to a 0.45 μm NC membrane (Sigma-Aldrich). The membrane was then blocked with 5% (w/v) milk in phosphate-buffered saline (PBS) for 2 h at 25°C and immersed in 1/5,000 rabbit polyclonal anti-human tissue-type plasminogen activator (tPA) antibody (Abcam) and 1/5,000 goat anti-rabbit HRP conjugated secondary antibody (Sigma-Aldrich) successively. The specific immunoreactive proteins were visualized by enhanced chemiluminescent (ECL)-solution (GE Healthcare).

### Quantification of rPA Accumulation in Plants

The rPA accumulation was measured by using an ELISA-based assay as previously described. The ELISA plate was coated with the leaf extracts (overnight at 4°C), blocked with 5% (w/v) PBS-made milk (1 h at 37°C), and incubated in 1/10,000 rabbit polyclonal anti-tPA antibody (2 h at 37°C) and 1/5,000 goat anti-rabbit HRP conjugated secondary antibody (2 h at 37°C) successively. After each step, the plate was washed three times with PBS buffer at 5-min intervals. The plate was re-filled with 3,3',5,5'-tetramethylbenzidine (TMB) substrate solution (Solarbio), incubated at 25°C for 15–30 min, and the reaction was halted by adding 1.0 M phosphoric acid. And then, OD values at 450 nm were read by using a microplate reader (Bio-Rad). A standard curve was generated with *Escherichia coli*–expressed rPA for each plate and the extracts from the empty vector (pCPMV-EV or pTMV-EV) infiltrated leaves were used as a negative control. The relative accumulation level was described as a fold change of the amount of rPA in the absence and presence of PI. The observed values were obtained from the mean of three independent biological replicates and were used to perform Student’s *t*-test.

## Results

### Co-Expression of PI Showing Significant Repression on RP Accumulation

The rPA expression vector is a CPMV (*Cowpea mosaic virus*) RNA2-based construct, which allows high-level RP expression in plants without viral replication (Sainsbury and Lomonossoff, 2008). And, both expression vectors for rPA and PIs have identical T-DNA backbone and transcriptional elements. Furthermore, they all are targeted to the secretory pathway guided by PR1b signal peptides (Figures 1A,B). The vectors were mixed in equal proportion (final OD_600_ of 0.6 for each) and then inoculated into *N. benthamiana* leaves by agroinfiltration (Figure 1C).

The individual agro-inoculum was infiltrated into different sectors of the same leaf (Figure 2A). The infiltrated leaves were then sampled at 5 day post-inoculation (dpi) and used for detecting the expression of recombinant rPA. Co-infiltration with rPA and PI expression vectors resulted in detectable immunoblot bands in western blot analysis with anti-rPA antibody, except for empty vector (Figure 2B). Bands of ca. 45.0 kDa had appeared in all the samples upon co-expression. Conversely, rPA expressed by *E. coli*, positive control, showed a major band with a position corresponding to 39.0 kDa. Since rPA contains two potential N-glycosylation sites at positions Asp-184 and -448 (Noble and McTavish, 1996), it indicated that this plant-derived rPA was glycosylated by post-translational processing. And, the weak bands were presumably due to proteolytic trimming or cleavage of recombinant rPA (Ny et al., 1984). The accumulation of rPA was then quantified using an ELISA assay. The results revealed a 65–84% decrease of rPA accumulation upon PI co-expression (Figure 2C), suggesting that co-expression of PI significantly represses the rPA accumulation in *N. benthamiana* leaves. Because vectors share similar expression elements, we speculate a competition between PI and rPA during co-expression. We speculate that PI overwhelms the rPA during the competition, therefore resulting in a repression of rPA expression. And, the competitive effects may be common among PIs because all three unrelated PIs show evident repression of rPA expression.

### Modulating PI Abundance in the PI-RP System Influencing the Outcome of the Competition

In an attempt to overcome the competitive effects, we kept the rPA construct (pCPMV-rPA) at a final OD_600_ value of 0.6 and gradually reduced the dose of *Sl*CYS8 in the co-inoculation mixtures. And, the mixes were agroinfiltrated into different sectors within the leaf, as described in Figure 3A. The results showed that the accumulation of recombinant rPA gently increased along with reducing the dose of *Sl*CYS8 inoculum in the co-inoculation mixtures (Figures 3B,C), indicating a negative correlation between the PI abundance of the PI-RP system and competitive effects. At the final OD_600_ value of 0.2, a ~60% increase of rPA accumulation was achieved (Figure 3C). Interestingly, rPA accumulation was similar to the control, with the rPA alone, at OD_600_ of 0.1 (Figure 3C). Because the protective effects of PIs are dose dependent *in vivo* (Grosse-Holz et al., 2018b), we speculate that the PI molecules in cells are not enough under this PI inoculation dose and therefore the repression overwhelms the protection, even though the repression has attenuated at this moment (OD_600_ value of 0.1), and thus no enhancement of rPA accumulation. Likewise, the protection is over the repression at OD_600_ value of 0.2, resulting in a slight increment of rPA. We tested *Nb*PR4 and *Hs*TIMP, respectively, and got similar results (Supplementary Figure S1), suggesting that modulating PI abundance in the PI-RP system influences the outcome of the competition (repression or increment of RP accumulation).

### Co-Expression of Replicating Vector With PI Overcoming Competitive Effects

The relative abundance of PI in the PI-RP system is critical for the result of the competition. If we use a viral replicating vector to express the RP instead of the non-replicating construct, the replication increases the RP level of the PI-RP system in inoculated cells, resulting in a relative decline of PI abundance without altering its absolute dose, thereby creating an optimal *in vivo* microenvironment for co-expression.

**Figure 4:**
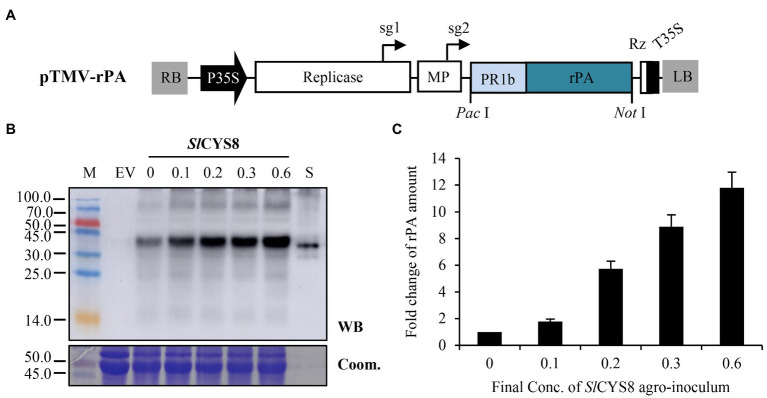
Co-expression of *Sl*CYS8 with a replicating vector overcomes repression on rPA accumulation. **(A)** The T-DNA region of the pTMV-rPA construct, a replicating TMV-mediated rPA expression vector. Replicase, RNA-dependent RNA polymerase of TMV; sg1 or sg2, subgenomic mRNA1 or mRNA2 promotor of the TMV; MP, movement-protein of TMV; Rz, ribozyme; T35S. *Pac*I and *Not*I, restriction enzymes. **(B)** Western blot analysis of crude leaf protein homogenate. The Coomassie-stained large subunit of Rubisco was used as a loading control (lower panel) and the polyclonal rabbit anti-tPA antibody was used to immunodetect rPA (upper panel). M, molecular weight marker (kDa); EV, empty vector, negative control; S, *E. coli*-expressed rPA, positive control. The numbers show the final OD_600_ value of the *Sl*CYS8 inoculum in the mixtures, while the final value of 0.6 is for the rPA construct. **(C)** ELISA assay of the relative rPA accumulation following *Sl*CYS8 co-expression. The data are presented as fold change of rPA amount in the absence or presence of *Sl*CYS8 and are determined from three separate batches of infiltration. The average value for treatment “0” is 69.3 μg/g +/− 1.2.

To verify this hypothesis, a replicating rPA expression vector (Figure 4A), pTMV-rPA, was constructed based on a TMV-derived vector (Lindbo, 2007). For comparison, we maintained the rPA expression cassette as same as that of the non-replicating vector, pCPMV-rPA. And, the final OD_600_ of the pTMV-rPA construct was kept at the same value as that of pCPMV-rPA at 0.6. The results showed that the rPA accumulation boosted along with the increment of *Sl*CYS8 dose (Figures 4B,C), showing a positive correlation between rPA accumulation and the dose of *Sl*CYS8 inoculum. Furthermore, the rPA accumulation was still boosted even at the maximal testing dose of the *Sl*CYS8, suggesting that the PI-RP system had an ignorable level of competitive effects while co-expression of replicating vector with PI. Similar results were obtained when testing *Nb*PR4 and *Hs*TIMP (Supplementary Figure S2). In addition, a 7- to 12-fold increase of rPA accumulation was achieved at the maximal testing dose of PIs (Figure 4C; Supplementary Figure S2), proving that it is feasible to overcome the competitive effects of the PI-RP system by using this *in vivo* approach.

## Discussion

Co-expression of PI is a promising strategy to improve the relatively low yields of RPs in plants by preventing RP from proteolytic degradation. Several PIs were demonstrated to guard RP against degradation *in vivo*, among which *Sl*CYS8 is the well-characterized one. However, only ~40% of increment on monoclonal antibody (mAb) was achieved upon *Sl*CYS8 co-expression (Robert et al., 2013). Furthermore, the co-expression of *Sl*CYS8 was reported to markedly decrease rather than increase α1ACT accumulation (Sainsbury et al., 2013). Conversely, the accumulation of three different RPs was enhanced ~7- to ~16-fold upon co-expression with *Sl*CYS8 when a mutant *Sl*CYS8 (*Sl*CYS8-Q47P), which lacks inhibitory activity (Jutras et al., 2016), was used as a negative control (Grosse-Holz et al., 2018b). Those pieces of evidence indicate that something behind the PI-RP co-expression system masks or obfuscates the practical potentials of a *Sl*CYS8 companion or a PI companion, in other words.

For exploring the issue, three unrelated PIs, including *Sl*CYS8, and rPA were chosen to establish the PI-RP co-expression system. To create an “authentic” PI-RP system, P19 and rPA (model RP) were expressed by a single construct (Figure 1A) instead of two separate constructs as previously described (Robert et al., 2013; Jutras et al., 2016; Grosse-Holz et al., 2018b). All the three PIs showed remarkedly repression on rPA accumulation in the leaves of *N. benthamiana* upon co-expression, although the strength of the repression differed among PIs (Figure 2). The results are similar to what was observed by Sainsbury et al. (2013). We speculate that there are competitive effects in the PI-RP system. This conclusion is drawn from three pieces of evidence. First, the vectors for both RP and PI share similar expression elements (T-DNA backbone, transcriptional elements, and signal peptides) but resulting in remarkable repression of RP (Figures 1A,B). Second, the repression can be attenuated by reducing the PI inoculum dose while the RP dose remains unchanged in the co-infiltration mixtures (Figure 3). Third, the repression reduces to an ignorable level when increasing RP abundance in the PI-RP system by replication (Figure 4). Moreover, the competitive effects may be common among the PIs because three testing PIs belong to different families.

The repression or increment of RP is the outcome of the competition between PI and RP. When PI overwhelms the RP during the competition, it results in a repression of RP expression (for example, α1ACT and rPA), otherwise, RP was found to increase (for example, antibody). Nevertheless, the modulating relative abundance of PI, whether *in vivo* or *in vitro*, can alter the outcome of the competition, indicating a negative correlation between the PI abundance and competitive effects. Grosse-Holz et al. (2018b) suggested that the protective effects of PI on RP are dose dependent *in vivo*. In the present study, RPs accumulate more with higher PI inoculum dose under an ignorable competition situation (Figure 4C; Supplementary Figure S2), also reasoning that it functions in a dose-dependent manner. Since both protective and competitive effects are related to PI abundance, modulating the relative abundance of PI seems to be a useful way to stimulate the potentials of the PI-RP system in the application. The repression can be attenuated by reducing the PI dose in the co-inoculation mixtures (Figure 2; Supplementary Figure S1). However, when repression was circumvented by this *in vitro* modulation, a companion PI lost most of its *in situ* protective effects due to inadequate dose at that moment (Figure 2; Supplementary Figure S1). Moreover, *A. tumefaciens* with a high density (above OD_600_ value of 1.0) results in leaf yellowing or wilting (Kagale et al., 2012). Therefore, the approach by modulating PI dose *in vitro* in co-inoculum has shortcomings in the application. Theoretically, an environment in which keeping the PI absolute abundance at a high level while the relative abundance in the PI-RP system low enough to avoid repression is useful in stimulating the protective potential of PIs. And, our studies showed that modulating PI abundance *in vivo* is more feasible than *in vitro* in terms of RP accumulation.

Sainsbury et al. (2013) speculated that competitive effects might occur during pre- and/or post-translational processes. In our study, the co-expression vectors have identical transcriptional elements (promoter and terminator), and increasing the abundance of RP mRNAs *in vivo* by viral replication reduces competitive effects. Those data suggest that the competitive effects may also occur during the translational processes, especially during protein synthesis. The PI mRNAs somehow are favored over the RP during translation, but this superiority is abolished when the abundance of RP mRNAs is dominant in the PI-RP system in cells.

## Data Availability Statement

The raw data supporting the conclusions of this article will be made available by the authors, without undue reservation.

## Author Contributions

SW designed the experiments and wrote the manuscript. SW and ZL supervised the research. JM and XD conducted the vector construction and agroinfiltration. All authors contributed to the article and approved the submitted version.

### Conflict of Interest

The authors declare that the research was conducted in the absence of any commercial or financial relationships that could be construed as a potential conflict of interest.
