# Supplementary material for: Co-expression With Replicating Vector Overcoming Competitive Effects Derived by a Companion Protease Inhibitor in Plants
Source: Front Plant Sci. 2021 Jun 17;12:699442. doi: 10.3389/fpls.2021.699442 (PMC8248793; doi:10.3389/fpls.2021.699442)
Supplement: Supplementary file 1 [file Data_Sheet_1.docx]

**Co-expression with replicating vector overcomes repressive effects** **derived by a companion protease inhibitor in plants**

Jiexue Ma^1^, Xiangzhen Ding^1, 2^, Zhiying Li^1, 2^ and Sheng Wang^1, 2, 3*^

^1^School of Life Science, Ningxia University, Yinchuan, China

^2^Key Laboratory of Ministry of Education for Protection and Utilization of Special Biological Resources in Western China, Yinchuan, China

^3^Key Laboratory of Modern Molecular Breeding for Dominant and Special Crops in Ningxia, Yinchuan, China

^*^Correspondence: wang_s@nxu.edu.cn

**Supplementary Figures**

**
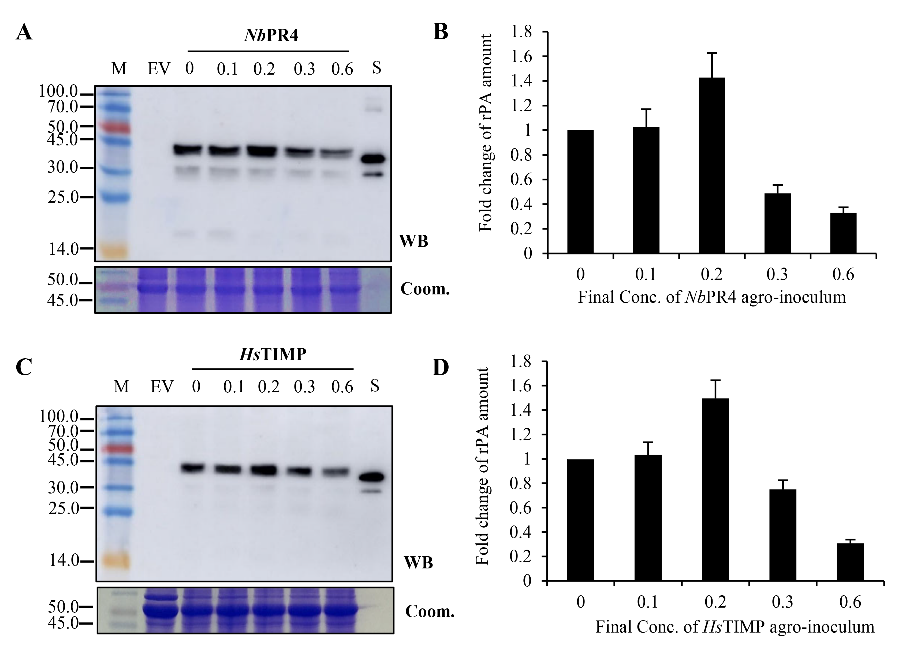
**

**Supplementary Figure 1. Modulating the dose of** ***Nb*PR4 or *Hs*TIMP inoculum in co-infiltration mixtures attenuates repression on rPA accumulation.** **(A) (C)** Western blot analysis of crude leaf protein homogenate. The Coomassie-stained large subunit of Rubisco was used as a loading control (lower panel) and the polyclonal rabbit anti-tPA antibody was used to immunodetect the recombinant rPA (upper panel). M, molecular weight marker (kDa); EV, empty vector, negative control; S, *E. coli*-expressed rPA, positive control. The numbers show the OD_600_ value of the PIs inoculum in the final mixtures. (**B**) (**D**) ELISA assay of the relative rPA accumulation following *Nb*PR4 or *Hs*TIMP co-expression. The data were presented as a ratio of rPA amount in the absence or presence of *Nb*PR4 or *Hs*TIMP and were determined from three separate batches of infiltrations.

**
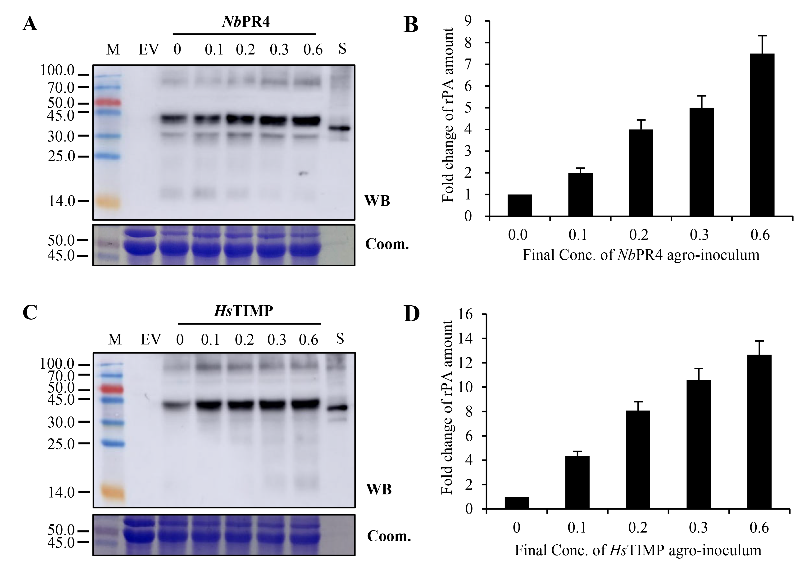
**

**Supplementary Figure 2. Co-expression of *Nb*PR4 or *Hs*TIMP with a replicating vector overcomes repression on rPA accumulation. (A) (C)**Western blot analysis of crude leaf protein homogenate. The Coomassie-stained large subunit of Rubisco was used as a loading control (lower panel) and the polyclonal rabbit anti-tPA antibody was used to immunodetect the recombinant rPA (upper panel). M, molecular weight marker (kDa); EV, empty vector, negative control; S, *E. coli*-expressed rPA, positive control. The numbers showing the OD_600_ value of PI inoculum in the final mixtures. **(B) (D)** ELISA assay of the relative rPA accumulation following *Nb*PR4 or *Hs*TIMP co-expression. The data were presented as a ratio of rPA amount in the absence or presence of PI and were determined from three separate batches of infiltration.
